# Indents

**Published:** 1919-08

**Authors:** 


### INDENTS

**☞Brief Articles of Technical or Scientific Value will receive notice and comment. Contributions invited.**

---

Method of Firm Attachment of Vulcanite to Aluminum Dentures. An aluminum plate with teeth attached by vulcanite forms a very light denture, comfortable to wear, and of little bulk. It is well known, however, that after being for some time in use the vulcanite is liable to separate from the aluminum, as the result of some chemical or electro-chemical action. A method of obviating a breakdown from this particular cause would probably lead many practitioners who have abandoned the combination to try it afresh. When the case is ready for rubber packing, the cleaned aluminum surface is coated with a thin solution of rubber in chloroform, and over this, when dry, is carefully packed a layer of weighted rubber, and the further filling of the mould space is completed with ordinary rubber. Those who have tried this method claim that the attachment between vulcanite and aluminum remains sound, and a stable union between the two is secured. A similar use of weighted rubber—of the metallic filling kind—was some years ago found very serviceable with the other metals in attaching teeth by vulcanite to "wire bar" lower cases; the resulting union was so very close and intimate that hardly anything short of filing or chiselling would effect their separation.—*Dental Record.*

---

What Is Nickel Silver? So-called German silver is a composition of nickel, copper and zinc in varying proportions. Its value in the arts depends on its color, lustre, hardness, tenacity, toughness, malleability, ductility, machining qualities, and resistance to alkalies and acids. Generally speaking, it is manufactured in three ways. The German method was to melt all the copper and two-thirds of the nickel and zinc in a graphite crucible; and then add the rest of the nickel and zinc. In the English method, all the copper, nickel and zinc are melted at one time, then zinc and copper are added, should a test show that the metal is porous; a fireclay pipe containng pitch is pushed into the metal to deoxide it. One American method is to melt a copper-nickel alloy and then gradually add the pre-heated zinc. "German silver" is now called nickel silver.—*The Crucible.*

---

INFANT MORTALITY RATES IN THE UNITED STATES. The New York Milk Commission has recently issued a report on the infant mortality rates of the different cities of the United States for the year 1918. This report shows that the infant death rate for New York City was 92 per thousand living births. Although the estimates show that the death rate for the country increased seven points last year, the milk commission believes these rates are remarkably low when all the elements conspiring against the baby are considered. The infant mortality rate for seventy-nine cities with populations under 50,000 was 97.2. The rate for thirty-eight cities of between 50,000 and 100,000 was 113.8, and that for forty-five cities of 100,000 population was 103.5. The average baby death rate for the registration area of the United States is 104. Twenty-two of the cities of 100,000 are above this average, and twenty-two are below. The rates for a number of the larger cities are reported as follows: Chicago, 104.3; Philadelphia, 126; Boston, 114.9; Baltimore, 147.8; Pittsburgh, 122.5; Buffalo, 121.5; Milwaukee, 108.2; Cincinnati, 104.1; Newark, N. J., 104.7; New Orleans, 123.3; Washington, D. C., 110.9; Jersey City, 118.7; Louisville, Ky., 117.3; Denver, 107.3; Syracuse, N. Y., 117.4; Birmingham, Ala., 133.5; Memphis, Tenn., 145; Scranton, Pa., 144.3; Richmond, Va., 146.3; Fall River, 161.3; Lowell, Mass., 159.1; Albany, N. Y., 107.4. Only three cities reported baby death rates below 50. These cities are all of the class below 50,000. Brookline, Mass., has the lowest rate, 35.4; Madison, Wis., is next with 38.1, and Pasadena, Calif., third, with 43.8.—*Journal A. M. A.*, August 2, 1919.

---

Anesthetics for the Aged. Dr. A. H. Miller, in the *Journal of A. M. A.* for August 2, 1919, says that he found a mortality of eight times more in 271 patients who were over fifty years of age as against 729 patients who were under fifty years of age.

---

The Science of Health. A correspondent asks whether we consider dentistry a branch of medicine. Dentistry is a branch of the science of health, as is also medicine. There is no conflict between these two branches of this department of science; nothing but the rivalry that may quite properly occur between sisters in the same family. These two branches, along with the other branches of this great science, will doubtless learn to work more and more in a spirit of harmony and co-operation as time goes by. In the meantime, no good purpose can be served by trying to tack dentistry on to medicine. It just can't be "tacked."

Possibly our correspondent attaches to the word "medicine" a much broader significance than it deserves under modern conditions of practice. A century ago the word embraced *all*, but today it includes only a *part*, and has come to .refer merely to certain well-defined branches of the science of health.

In the practice of medicine, so many specialties have developed, that some kind of a reorganization of medical education is demanded by the medical men themselves. Why not a comprehensive plan that would include all branches of the science of health? The suggestion of a common two years' course in the pure science subjects, followed by a specialized course in the branch desired, is being seriously considered in some universities, and would be a long step forward. For instance, following the first two years, in addition to a specialized course for the general practitioner, there might be courses in surgery, dentistry, physical therapy, eye, ear, nose and throat, public health service, and other important branches. Upon graduation all would have equal recognition, and each would be trained to co-operate with the other, with a view to the best possible service. We believe some such plan (which is neither new nor original) will be in operation within ten years, and will do much to eliminate the present-day monopolistic provincialism that is so frequently manifested, and which so seriously interferes with efficient health service.—W. Seccombe, in *Oral Health.*

---

Strength of Epinephrin for Local Anesthesia.

*To the Editor*—Please give me the following information:

1. What strength of epinephrin (adrenalin) should be used in injecting tonsils for local anesthesia?

2. What are the dangers of strong solutions of epinephrin, aside from the secondary hemorrhage?

3. Would 1 dram of 1:1,000 solution of epinephrin to the ounce of 1:500 cocain, and using 2 drams in each tonsil, be dangerous? Please omit my name.—B. E. M.

*Answer.*—1. From 10 to 15 drops of the 1:1,000 solution of epinephrin per ounce of anesthetic solution is the strength usually employed.

2. The chief danger of strong solutions lies in the possibility of injecting it inadvertently into a vein, in which case 1 c.c. of a 1:1,000 solution might produce most alarming and even fatal results from overwhelming effect on the circulation. In subcutaneous infiltration, there is the additional danger of necrosis from excessive vasoconstriction; but this probably does not apply to as vascular a tissue as that of and around the tonsil.

3. The addition of 1 dram of a 1:1,000 solution of epinephrin to the ounce of 1:500 cocain would probably not be very dangerous; but the concentration is greater than that usually employed, and, when the solution is used as freely as 2 drams per tonsil, it might not be entirely devoid of danger.—*Journal A. M. A.*, July 26, 1919.

---

Canadian Foundation for Dental Research. For years the dental profession of Canada has labored without a systematized method of solving the manifold problems of modern dentistry. Men have struggled, single-handed, to unravel the mysteries surrounding subjects vital to the dental profession and the human race, and, because of insufficient funds, their earnest efforts have not been wholly successful. During the past few years, however, the wonderful work of Canadian dentists, at home and overseas, has added to the prestige of Canadian dentistry, and, as a result, a new impetus has been given to dental research in Canada. The Dental Research Committee of Canada has been organized to have representatives from each province of the Dominion.

Interest in dental research has been shown not only by the graduates, but also by the undergraduate body. During the recent Victory Loan flotation a few students of the Royal College of Dental Surgeons of Ontario invested in bonds, but the majority, who were unable to purchase a bond, wished to help in floating the great loan. At a mass meeting of the students ideas were exchanged and suggestions advanced. As a result of this meeting the students unanimously decided to raise as a minimum by undergraduate subscription, the sum of five hundred dollars, to be invested in Victory Bonds, to be the nucleus of a fund for the advancement of dental research. Members of the faculty of the college by generous subscription and a special grant from the Students' Executive, increased the amount to fifteen hundred dollars, which was duly invested in Victory Bonds.

In response to this evidence of student interest in research work, the Dental Research Committee of Ontario subsequently invited the undergraduate body to elect a representative to be a member of the Research Committee. —*Oral Health.*

What Did I Do to This Patient? A young lady about nineteen years old came to me for the insertion of several fillings. None of the work was difficult. I saw immediately that she was very nervous—remarkably so. Her mother, who was with her, said she had been always delicate. I anticipated trouble and made up my mind that something must be done right on the start or I would be up against it. I hastily examined her teeth with some difficulty. She then partially raised up and sat a little forward in the chair, I gently pushed her back and inclined the chair backward about 45 degrees. I put my left hand gently on her forehead and very lightly smoothed back the hair lying on her forehead. At the same time I said, "Now calm yourself and go to sleep, you will not be hurt." I waved my right hand in a playful way through the air like I had seen a hypnotist do once, laughing as I did so. She smiled and closed her eyes and in a moment I noticed her muscles relax seemingly all over the body. I thought it merely a voluntary relaxation, but when I spoke to her and she did not answer, I gently shook her arm and still no response. I pride myself of keeping a pretty good equilibrium through most ordeals, but I now began to think my patient's nervousness had been transmitted to myself. After trying several times to bring her to consciousness I lowered her to a lying position. The mother came forward rather excitedly but as nonchalantly as possible I gave her to understand that this was an everyday occurrence. I told her that her daughter would be all right in a moment, but it was fully five minutes before my patient opened her eyes.

The question is, What did I do to this patient? Did she faint? I do not think so, because instead of paling, as is usual in cyncope, the patient's face became slightly flushed. After this episode I finished the operation without the least bit of trouble. I am still wondering what I did to that patient. I do not believe in hynotism, as applied this way, and certainly I have no such power.—*Dental Digest.*

